# Radiological Anatomy of the Shoulder, Elbow and Carpal Joints in Southern Giant Pouched Rats (*Cricetomys ansorgei*)

**DOI:** 10.1155/vmi/3532418

**Published:** 2026-07-23

**Authors:** Veronica Masawe, Modesta Makungu

**Affiliations:** ^1^ Department of Veterinary Medicine and Public Health, College of Veterinary Medicine and Biomedical Sciences, Sokoine University of Agriculture, P. O. Box 3021, Morogoro, Tanzania, suanet.ac.tz; ^2^ Department of Veterinary Surgery and Theriogenology, College of Veterinary Medicine and Biomedical Sciences, Sokoine University of Agriculture, P. O. Box 3020, Morogoro, Tanzania, suanet.ac.tz

**Keywords:** anatomy, diagnostic imaging, giant rat, joint, radiography, thoracic limb

## Abstract

Radiography is frequently used for the evaluation of the musculoskeletal system in domestic and wild animal species. The aim of the study was to document the radiological anatomy of the shoulder, elbow and carpal joints and to provide length measurements of selective forelimb bones in southern giant pouched rats. Forelimb radiographs were obtained in seven adult male and female southern giant pouched rats under general anaesthesia. All rats had a well‐developed clavicle, coracoid process and acromion. The minor and major tubercles were located further distally relative to the head of the humerus. The medial epicondyle and lateral supracondylar crest were very prominent. The supracondylar foramen was present in all rats. The radius was relatively smaller than the ulna, and the interosseous space was wide. The carpus had eight carpal bones, and two rudimentary digits were visualised on the palmar side of the manus. Additionally, five slender, widely spaced metacarpal bones, were seen with the third metacarpal bone being relatively the longest and the first metacarpal bone being the shortest. Shoulder joint dysplasia was identified in one female southern giant pouched rat. Radiographic examination of the thoracic limb in southern giant pouched rats allowed evaluation of bones forming the shoulder, elbow and carpal joints. Current information will contribute to the understanding of rodent anatomy and will be useful for scientists researching the forelimb of rodents and related species. Additionally, this information will augment the application of radiography and other related diagnostic imaging modalities in rodents and hence will enhance their welfare.

## 1. Introduction

Radiography is an imaging modality that is currently widely used in companion and exotic animals for the diagnosis and evaluation of healing progress of various diseases and conditions, selective breeding programmes and prepurchase examinations [1, 2]. It is an imaging technique that uses X‐radiation to visualise internal structures [3]. Therefore, appropriate training and awareness of the possible hazards are important for the safety of the patient, personnel and X‐ray equipment [4].

There are mainly two types of radiography systems currently used in veterinary practices: the conventional film‐screen radiography and digital radiography system [5]. The latter includes computed radiography (CR) and direct radiography (DR) [5]. Depending on detectors/conversion systems, the DR system can further be classified into indirect and direct conversion systems [5]. The introduction of DR systems has significantly revolutionised the veterinary practice in terms of production efficiency and transmission [3].

Radiography is still considered the best imaging modality for the assessment of bone structures in veterinary patients [2]. It has the ability to recognise and typify various bone lesions such as aggressive versus nonaggressive and to assess bone response to treatment [2]. Nevertheless, a good understanding of the radiographic technique and radiological anatomy of an area of interest in a particular species is of paramount importance.

Southern giant pouched rats are wild rodents belonging to the family Nesomyidae. They are primarily found in the savanna of East and Southern Africa, including Tanzania [6]. At present, they are widely kept in captivity for biomedical research, detection of mines and as pet animals [7–10]. Bone diseases/conditions involving the forelimb such as fractures, exostoses and neoplasia have been observed in wild rodents [11] similar to companion animals.

This study documents the radiological anatomy of the shoulder, elbow and carpal joints and provides length measurements of selective forelimb bones in southern giant pouched rats for clinical, teaching, biomedical and related research.

## 2. Materials and Methods

### 2.1. Study Area

The study was done at the Department of Veterinary Surgery and Theriogenology in the College of Veterinary Medicine and Biomedical Sciences of the Sokoine University of Agriculture. The study was approved by the College of Veterinary Medicine and Biomedical Sciences (CVMBS) Research, Publication and Innovation Committee.

### 2.2. Southern Giant Pouched Rats

Seven adult intact male and female southern giant pouched rats were included in this study. Of the seven southern giant pouched rats, five were males with minimum and maximum weights of 1.02 kg and 1.35 kg, respectively (mean: 1.19 ± 0.14 kg). There were two female with a mean weight of 1.12 ± 0.11 kg (range: 1.04–1.20 kg). The overall mean weight of all southern giant pouched rats was 1.17 ± 0.13 kg (range: 1.02–1.35 kg). All southern giant pouched rats were caught from their native environment and were healthy based on clinical examination.

### 2.3. Chemical Restraint

In all southern giant pouched rats, imaging of the right forelimb was performed after the administration of a combination of xylazine (Interchemie Werken, Holland) and ketamine hydrochloride (Troikaa Pharmaceuticals Ltd., India; Rotexmedica, Germany). The two drugs were combined in a single syringe (2 mL: 1‐inch, 23‐gauge needle) and used for chemical restraint. A combination of xylazine (5 mg/kg body weight) and ketamine hydrochloride (50 mg/kg body weight) was injected intramuscularly into the quadriceps femoris muscle. The onset of anaesthesia took 2 to 3 min with a duration of effect of approximately 120 min. The primary vital signs were closely monitored in all southern giant pouched rats until the animals had completely recovered from anaesthesia. A combination of xylazine and ketamine hydrochloride was chosen for its efficacy in southern giant pouched rats [12–16].

### 2.4. X‐Ray Equipment

Forelimb radiographic examinations were done after the administration of general anaesthesia. Roller 30 X‐ray machine (SMAM X‐ray Equipments, Italy) was utilised at 100‐cm source‐to‐image distance, and nongrid exposures of 46–48 kVp and 2.5 mAs were used. Image acquisition in all southern giant pouched rats was performed using a CR system (Colenta HighCap Xr, Fuji Film Corporation).

### 2.5. Radiographic Views and Positioning

**FIGURE 1:**
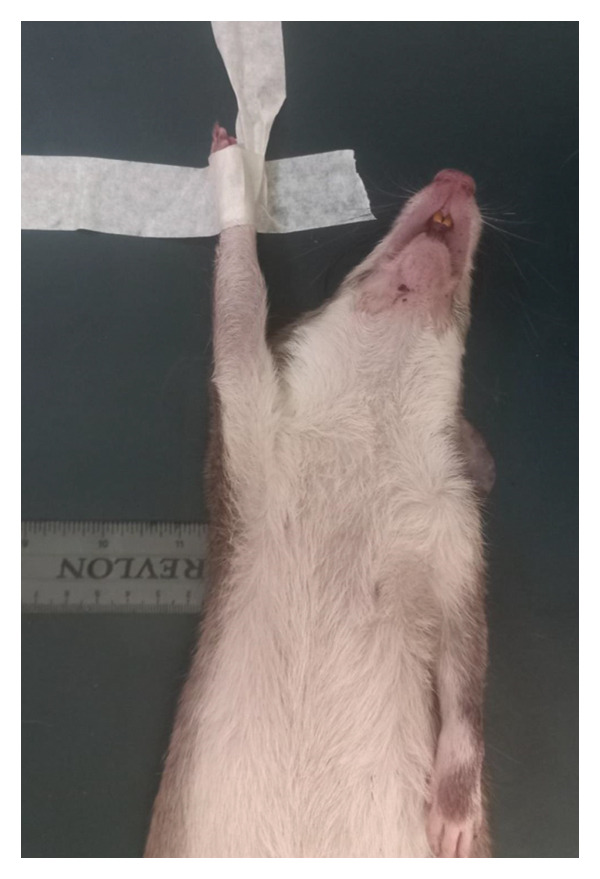
Photograph of an adult intact southern giant pouched rat illustrating positioning for the caudocranial (CdCr) radiographic projection of the right forelimb. The southern giant pouched rat is positioned in dorsal recumbency, and the right forelimb is extended cranially and secured on the cassette with masking tape. The left forelimb is extended caudally.

**FIGURE 2:**
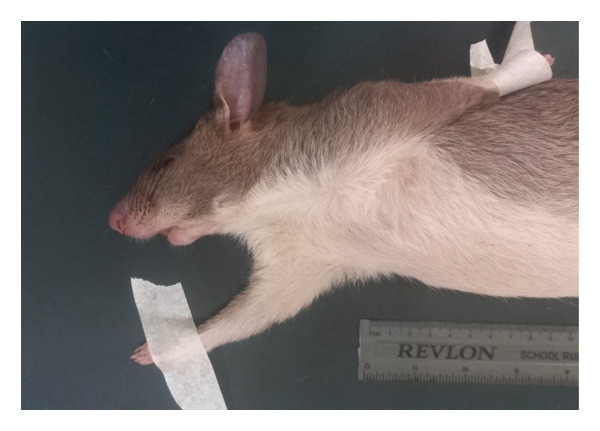
Photograph of an adult intact southern giant pouched rat illustrating positioning for the mediolateral (ML) radiographic projection of the right forelimb. The southern giant pouched rat is positioned in the right lateral recumbency, and the right and left forelimbs are extended cranially and caudally, respectively, and secured on the cassette with masking tape.

**FIGURE 3:**
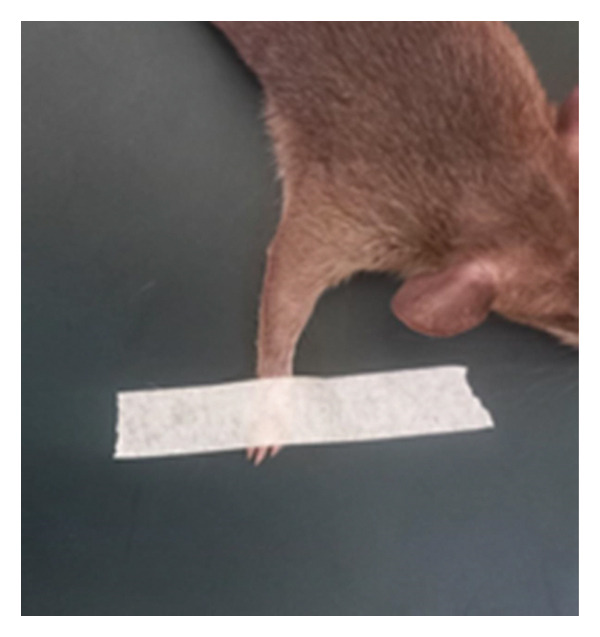
Photograph of an adult intact southern giant pouched rat illustrating positioning for the dorsopalmar (DPa) radiographic projection of the right manus. The southern giant pouched rat is in a sternal recumbency, the right forelimb is extended cranially, and the manus is secured on the cassette with masking tape.

Two projections of the right forelimb were taken: the mediolateral (ML) and caudocranial (CdCr) projections. Dorsal recumbency with the right forelimb extended cranially and secured with masking tape was applied for a CdCr projection (Figure 1), whereas a right lateral recumbency with the right elbow joint semiextended and held in place with masking tape was applied for the ML projection (Figure 2). The beam was centred at the elbow joint, and collimation encompassed proximally the shoulder and distally the manus for the CdCr and ML projections. Additionally, a dorsopalmar (DPa) projection of the manus was acquired with southern giant pouched rats in sternal recumbency and the limb extended cranially with the manus secured in place with masking tape (Figure 3).

### 2.6. Evaluation of Images

All images of the right forelimb were assessed using an HP Elite Display E221 (Qisda [Suzhou] Co. Ltd, China) monitor with a Colenta Dx Easy Imaging AQS Version 2.10.31 (COLENTA Labortechnik GmbH & Co KG, Austria). Radiographs were assessed based on the radiographic features, and length measurements of selected forelimb bones were performed. The ML projection was used for length measurements of the antebrachial bones, humerus and rudimentary digits, whereas for phalanges and metacarpus, the DPa projection was used for length measurements. Length measurements of the forelimb bones were done from the proximal extremity to the distal extremity of respective bones.

### 2.7. Data Analysis

Length measurements of the forelimb bones and weights of southern giant pouched rats were entered into Microsoft Excel. Range, standard deviation and mean were calculated using Epi Info Version 7.2.6.0 statistical software.

## 3. Results

### 3.1. Shoulder Joint

**FIGURE 4:**
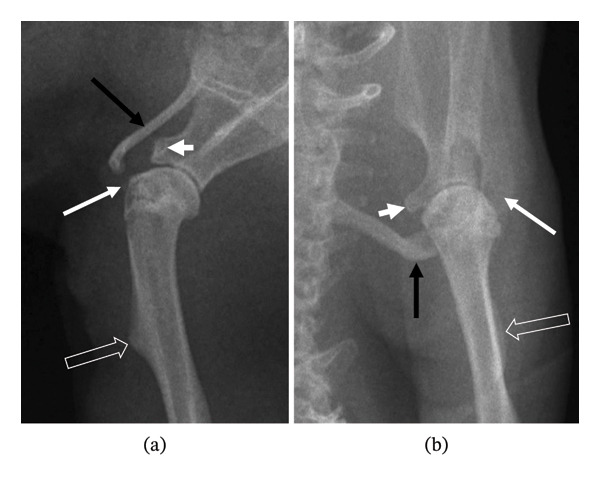
Mediolateral (a) and caudocranial (b) radiographic projections of the shoulder joint of an adult male southern giant pouched rat. Note the presence of a well‐developed clavicle (black arrows), coracoid process (short white arrows) and the hamate process (long white arrows). The minor and major tubercles are distal to the humeral head. The pronounced deltoid tuberosity (white open arrows) appears fairly triangular and rectangular on the mediolateral (a) and caudocranial (b) radiographic projections, respectively.

All southern giant pouched rats had a well‐developed clavicle (Figure 4). On the ML projection, the slender and curved clavicle was seen cranial to the shoulder joint at the distal half of the scapula (Figure 4a), with its sternal extremity superimposed on the scapular cranial margin (Figure 4a). The clavicle was seen medial to the shoulder joint just distal to the humeral head and the minor tubercle on the CdCr projection (Figure 4b). It appeared sigmoid‐shaped with the sternal and acromial extremities superimposed on the cervical spine and medial half of the humerus, respectively (Figure 4b). The acromial and sternal extremities of the clavicle were seen to articulate with the hamate process and *manubrium sterni*, respectively (Figure 4).

**FIGURE 5:**
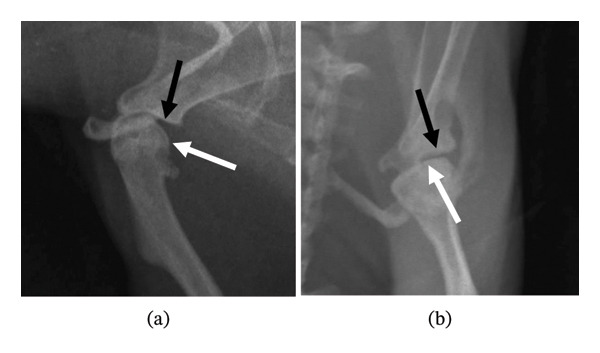
Mediolateral (a) and caudocranial (b) radiographic projections of the shoulder joint of an adult female southern giant pouched rat with an incidental shoulder joint dysplasia. Note the flattening of the humeral head (white arrows) and the glenoid cavity (black arrows). There is also widening of the glenohumeral joint space.

A greater part of the hamate process was superimposed on the humeral proximal extremity, with its distal end projected cranial to the major and minor tubercles on the ML projection (Figure 4a). At the scapular craniodistal margin, a curved and conspicuous coracoid process was visualised (Figure 4a). On the CdCr projection, the coracoid process (Figure 4b) was located medial to the shoulder joint. The well‐developed, laterally situated hamate process extended further distally than the glenoid cavity, with its distal end superimposed on the humeral proximal extremity (Figure 4b). The glenoid cavity appeared concave on the CdCr and ML projections (Figure 4). On the two projections, the humeral head was seen rounded and the minor and major tubercles proximally did not reach the humeral head (Figure 4). The two tubercles appeared almost flattened on the CdCr projection (Figure 4b). Incidental shoulder joint dysplasia was seen in one female southern giant pouched rat (Figure 5). Measurements of the humerus are indicated in Table 1.

**TABLE 1:** Length measurements (mm) of selected forelimb bones in southern giant pouched rats.

Bone	No. of animals	Range	Mean ± SD
Humerus	7	44.0–52.0	48.3 ± 2.7
Radius	7	45.0–50.0	47.1 ± 1.8
Ulna	7	57.0–63.0	59.6 ± 2.3
MCI	7	3.0–4.0	3.9 ± 0.4
MCII	7	9.0–12.0	10.3 ± 1.1
MCIII	7	12.0–14.0	12.9 ± 0.9
MCIV	7	10.0–13.0	11.7 ± 1.1
MCV	7	7.0–8.0	7.9 ± 0.4
PIDI	7	3.0–4.0	3.7 ± 0.5
PIDII	7	3.0–6.0	5.6 ± 1.1
PIDIII	7	6.0–8.0	6.6 ± 0.8
PIDIV	7	6.0–7.0	6.1 ± 0.4
PIDV	7	5.0–6.0	5.4 ± 0.5
PIIDII	7	1.0–3.0	2.0 ± 0.8
PIIDIII	7	2.0–3.0	2.6 ± 0.5
PIIDIV	7	2.0–3.0	2.4 ± 0.5
PIIDV	7	2.0–3.0	2.1 ± 0.4
PIIIDI	7	2.0–2.0	2.0 ± 0.0
PIIIDII	7	3.0–3.0	3.0 ± 0.0
PIIIDIII	7	3.0–3.0	3.0 ± 0.0
PIIIDIV	7	3.0–3.0	3.0 ± 0.0
PIIIDV	7	2.0–2.0	2.0 ± 0.0

*Note:* MC, metacarpal; PI, proximal phalanx; PII, middle phalanx; PIII, distal phalanx; *D*, digit.

### 3.2. Elbow Joint

**FIGURE 6:**
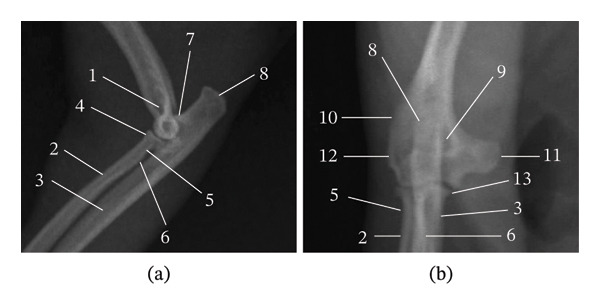
Mediolateral (a) and caudocranial (b) radiographic projections of the elbow joint of an adult female southern giant pouched rat. 1: supracondylar foramen, 2: radius, 3: ulna, 4: head of the radius, 5: radial neck, 6: radial tuberosity, 7: anconeal process, 8: *Tuber olecrani*, 9: olecranon fossa, 10: lateral supracondylar crest, 11: medial epicondyle, 12: lateral epicondyle, 13: medial coronoid process of the ulna.

On the ML projection, an ovoid radiolucent area, elongated proximodistally, was seen in the distal extremity of the humerus representing the supracondylar foramen (Figure 6a). The radius was relatively smaller than the ulna, and the interosseous space was wide (Figure 6a). The radial head was distinct with a slightly concave articular fovea. The former was well defined from the radial body (Figure 6a) as a result of a relatively long distinctive radial neck. The olecranon had concave and convex cranial and caudal margins, respectively, with a short anconeal process (Figure 6a). Proximally, the olecranon presented a slightly cranially directed and square‐shaped tuber olecrani (Figure 6a). Measurements of the radius and ulna are provided in Table 1.

On the CdCr projection (Figure 6b), a very pronounced medial epicondyle, compared to its lateral counterpart, was visualised. An ovoid radiolucent area elongated mediolaterally was seen in the distal extremity of the humerus representing the olecranon fossa (Figure 6b). The distal humerus had a pronounced lateral supracondylar crest and medial trochlea lip (Figure 6b). The medial coronoid process appeared pointed (Figure 6b).

### 3.3. Carpal Joint

**FIGURE 7:**
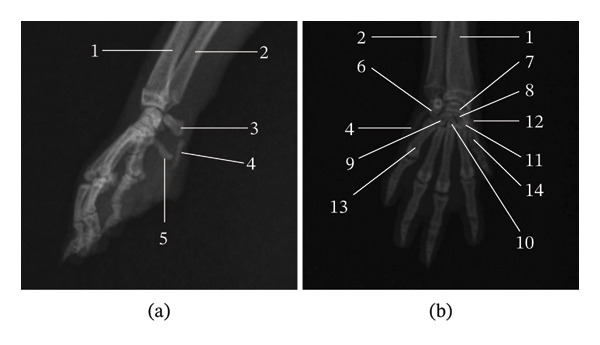
Mediolateral (a) and dorsopalmar (b) radiographic projections of the manus of an adult male southern giant pouched rat. Note the presence of eight carpal bones and five metacarpal bones and digits. 1: radius, 2: ulna, 3: accessory carpal bone, 4: rudimentary digit palmarodistal to the accessory carpal bone, 5: rudimentary digit palmar to the first metacarpal bone, 6: ulnar carpal bone, 7: intermedioradial carpal bone, 8: central carpal bone, 9: fourth carpal bone, 10: third carpal bone, 11: second carpal bone, 12: first carpal bone, 13: fifth metacarpal bone, 14: first metacarpal bone.

The distal extremities of the antebrachial bones were well‐developed, and their physeal scars were transverse (Figure 7). The carpus consisted of eight bones, namely, the ulnar, intermedioradial, accessory, central, fourth, third, second and first carpal bones (Figure 7). On the ML projection, the radius was situated more cranial than the ulna (Figure 7a). The accessory carpal bone was elongated dorsopalmarly and appeared bulbous (Figure 7a). Rudimentary digits were visualised on the palmar side of the first metacarpal bone and palmarodistal to the accessory carpal bone in all southern giant pouched rats (Figure 7a). The rudimentary digit palmarly to the first metacarpal bone was elongated dorsopalmarly, whereas the rudimentary digit palmarodistal to the accessory carpal bone was elongated proximodistally (Figure 7a). The rudimentary digit palmarodistal to the accessory carpal bone was visualised on the lateral side of the fifth metacarpal bone on the DPa projection (Figure 7b). The rudimentary digit palmarly to the first metacarpal bone was not clearly visualised (Figure 7b) as a result of its superimposition on the second and first carpal bones. Five widely spaced and slender metacarpal bones were seen (Figure 7). The third digit and metacarpal bone were the longest (Figure 7). Measurements of the metacarpal bones and phalanges are indicated in Table 1.

## 4. Discussion

Radiographic examination of the forelimb in southern giant pouched rats allowed evaluation of bones forming the shoulder, elbow and carpal joints, which is important in understanding the functional anatomy of the shoulder, elbow and carpal joints in this species. Additionally, this information is valuable in comparative anatomical studies and research involving rodents. Clinically, information from this study will aid clinicians in recognition of various orthopaedic conditions involving the forelimb, such as fractures, neoplasia, dysplasia and nutritional secondary hyperparathyroidism.

Southern giant pouched rats are terrestrial animals capable of climbing [17–19] and swimming [17]. Additionally, they use their forelimbs for digging [18] and holding food during feeding [17]. These activities are most likely accomplished in southern giant pouched rats as a result of the presence of powerful flexor muscles and high flexibility of their forelimbs. During climbing, feeding and digging, the forelimb in southern giant pouched rats is commonly held in flexion, which necessitates robust flexor muscles.

The existence of robust flexor muscles of the forelimb in southern giant pouched rats was demonstrated by the prominent medial epicondyle and well‐developed deltoid tuberosity. The forelimb digital and carpal flexor muscles originate at the medial epicondyle [20, 21], and the power of these muscles was pointed out by the conspicuous medial epicondyle. The latter was also reported to be prominent in other mammalian species with climbing and digging abilities and that use their forelimb as a grasping tool [22–25]. The deltoid tuberosity is the site for the insertion of the muscle deltoideus that flexes the shoulder joint [20, 21, 24]. Again, the well‐developed deltoid tuberosity in southern giant pouched rats demonstrates the strength of the deltoideus muscle in shoulder joint flexion.

In a study, which involved comparison of the morphology of the humerus in mole rats, the deltoid tuberosity and medial epicondyle were observed to be larger in scratch‐digger African mole rats than in chisel‐tooth digger African mole rats [24]. The presence of a pronounced medial epicondyle and deltoid tuberosity seen in southern giant pouched rats is similar to that seen in Norway albino rats [20], scratch‐digger African mole rats [24] and greater cane rats [25]. However, the medial epicondyle in southern giant pouched rats is relatively more pronounced than the one seen in scratch‐digger African mole rats and greater cane rats. This is presumably associated with additional climbing ability seen in southern giant pouched rats.

Contrarily, the medial epicondyle and deltoid tuberosity in guinea pigs [26, 27] and rabbits [27–29] are less pronounced and most likely are associated with a relatively increased degree of terrestriality [27]. This is further supported by the position of the proximal margin of the major tubercle proximal to the humeral head in guinea pigs [26, 27] and rabbits [27, 29, 30], which heightens the extensor function of the forelimb, which facilitates fast running [27, 31]. The distally situated major tubercle relative to the humeral head seen in southern giant pouched rats is analogous to mole rats [24, 32] and Norway albino rats [20]. The visualisation of the supracondylar foramen in southern giant pouched rats is similar to an osteological report in giant pouched rats [33], which is different from reports in guinea pigs [26, 27], greater cane rats [25] and rabbits [27, 28]. The supracondylar foramen accommodates and protects the brachial artery and median nerve from pressure injury [27].

Flexibility of the forelimb in southern giant pouched rats is pointed out by the well‐developed lateral supracondylar crest, clavicle, coracoid process, ulna, five widely spaced metacarpal bones and the presence of rudimentary digits. The lateral supracondylar crest in southern giant pouched rats provides the origin for the muscle supinator [20]. The latter supinates the manus and antebrachium, and its strength is shown by the well‐developed lateral supracondylar crest. The presence and well‐developed clavicle is associated with shoulder mobility [31]. In a study that compared the forelimb of some African Viverridae, the clavicle was seen in species with their primary locomotor category involving arboreal and terrestrial walking and jumping [31]. The muscle coracobrachialis takes its origin from the coracoid process [20], which is an adductor of the shoulder joint. The power of this muscle in adduction of the shoulder is demonstrated by the prominent coracoid process. The well‐developed ulna seen in southern giant pouched rats permits pronation and supination of the radius by acting as a pivot [22, 23, 25]. The rudimentary digits and widely spread out metacarpal bones in southern giant pouched rats play a role in the grasping function of the manus [25].

The visualisation of a well‐developed lateral supracondylar crest, clavicle, coracoid process and the relatively larger ulna compared with the radius seen in this species is analogous to mole rats [32] and Norway albino rats [20]. Greater cane rats have a prominent clavicle and coracoid process; however, their lateral supracondylar crest is inconspicuous and their antebrachial bones are more or less of similar size [25]. This indicates that the forelimb in the greater cane rat is not as flexible as in the southern giant pouched rat, mole rat and Norway albino rat. The coracoid process and clavicle in rabbits and guinea pigs are pronounced; however, the antebrachial bones are almost of the same size with a reduced interosseous space and lateral supracondylar crest [28, 30]. This further points out reduced flexibility of the forelimb relative to greater cane rats, mole rats, Norway albino rats and southern giant pouched rats, which favours terrestriality.

In this study, all southern giant pouched rats had eight carpal bones, as reported in studies on the mole rat [32], greater cane rat [25], Norway albino rat [20] and other burrowing rodents [34]. This finding is unlike that in the rabbit, which has been reported to have nine carpal bones [28, 30]. This is due to the presence of unfused intermediate and radial carpal bones in rabbits.

The two rudimentary digits, which were seen on the palmar side of the carpus in this species, have also been seen in greater cane rats [25], Norway albino rats [20], mole rats [24, 32] and in other burrowing rodents [34]. In previous rodent studies, these rudimentary digits were referred to as ‘bony structure’ [24] and ‘sesamoids’ (‘falciformis’ and ‘prepollex’) [20, 32, 34]. In fossorial rodents, these rudimentary digits aid during the grasping action of the manus in loosening/breaking up the soil during burrowing [24]. Therefore, it is most likely that these rudimentary digits in southern giant pouched rats not only assist during the grasping action of the manus for loosening the soil during burrowing but also during feeding. In a study that compared the forelimb of mole rats, the two rudimentary digits were found to be prominent in scratch‐digging species [24]. Furthermore, in burrowing South American octodontoid rodents, the rudimentary digit palmar to the first metacarpal bone was relatively prominent in the most specialised scratch‐digger rodent [34]. The presence of these rudimentary digits is unlike reported studies in the rabbit [28, 30]. The slender and widely spaced metacarpal bones seen in southern giant pouched rats represent a functional compromise of the manus, most likely as a result of being used for climbing, digging and feeding. Widely spread out metacarpals offer huge areas that enhance efficiency of the muscles that control digit movements [22]. In more specialised burrowing rodents, metacarpal bones are stout with a short and broad fifth metacarpal bone [24, 34].

Shoulder joint dysplasia, which was observed in a female southern giant pouched rat, has also been reported in the Dachshund and other breeds of dogs [35]. Dysplasia of the shoulder joint occurs as a result of a fault in the development of the ossification centre of the caudal portion of the humeral head, which results in the collapse of the caudal humeral head [35], as it was observed in this study. Furthermore, the glenoid cavity will fail to develop its concave articular surface due to the absence of support provided by the humeral head, resulting in incongruity of the shoulder joint [35].

Future radiographic studies of the manus in splayed‐toe lateral and oblique projections are recommended to improve visualisation of individual digits and metacarpal bones so as to enhance their anatomical detail. This is because the ML projection of the manus is limited by superimposition of the digits and metacarpal bones. Additionally, further studies that provide cross‐sectional anatomy of the forelimb and those which optimise visualisation of the associated soft tissue, such as magnetic resonance imaging, ultrasonography and computed tomography, are recommended in southern giant pouched rats.

## 5. Conclusions

The present study has provided the radiological anatomy of the shoulder, elbow and carpal joints in southern giant pouched rats. Additionally, this study has highlighted functional aspects of the associated structures. Moreover, comparison has been made with other rodents and the rabbit. The current information on the forelimb of southern giant pouched rats will contribute to the understanding of rodent anatomy and will be useful for scientists researching the forelimb of rodents and related species. Additionally, these findings will augment the application of radiography and other related diagnostic imaging modalities in southern giant pouched rats and other related species, thereby enhancing their welfare.

### 5.1. Limitation of the Study

The limitation of this study is the smaller sample size of southern giant pouched rats, which may affect the radiographic measurement of the forelimb.

## Author Contributions

Veronica Masawe: data curation, formal analysis, funding acquisition, investigation, methodology, project administration, visualisation, writing–original draft and writing–review and editing; Modesta Makungu: conceptualisation, supervision and writing–review and editing.

## Funding

This research was funded by the Sokoine University of Agriculture.

## Conflicts of Interest

The authors declare no conflicts of interest.

## Data Availability

The data that support the findings of this study are available from the corresponding author upon reasonable request.
